# Green synthesis and characterization of iron-oxide nanoparticles using Moringa oleifera: a potential protocol for use in low and middle income countries

**DOI:** 10.1186/s13104-022-06039-7

**Published:** 2022-04-25

**Authors:** Henry Fenekansi Kiwumulo, Haruna Muwonge, Charles Ibingira, Michael Lubwama, John Baptist Kirabira, Robert Tamale Ssekitoleko

**Affiliations:** 1 Department of Medical Physiology, Makerere University, Kampala, Uganda; 2 Department of Human Anatomy, Makerere University, Kampala, Uganda; 3 Department of Mechanical Engineering, Makerere University, Kampala, Uganda; 4 Habib Medical School, Islamic University in Uganda (IUIU), Kampala, Uganda

**Keywords:** Green synthesis, Bio-compatible, Iron oxide nanoparticles, Moringa oleifera, LMICs, UV–vis, X-ray diffraction, Scanning Electron Microscope, Energy Dispersive X-ray

## Abstract

**Objective:**

Green synthesized iron(III) oxide (Fe_3_O_4_) nanoparticles are gaining appeal in targeted drug delivery systems because of their low cost, fast processing and nontoxicity. However, there is no known research work undertaken in the production of green synthesized nano-particles from the Ugandan grown Moringa Oleifera (MO). This study aims at exploring and developing an optimized protocol aimed at producing such nanoparticles from the Ugandan grown Moringa.

**Results:**

While reducing ferric chloride solution with Moringa oleifera leaves, Iron oxide nanoparticles (Fe_3_O_4_-NPs) were synthesized through an economical and completely green biosynthetic method. The structural properties of these Fe_3_O_4_-NPs were investigated by Ultra Violet–visible (UV–Vis) spectrophotometry, X-ray diffraction (XRD), energy dispersive X-ray spectroscopy (EDX) and scanning electron microscopy (SEM). These nanoparticles exhibited UV–visible absorption peaks at 225 nm (nm) for the sixth dilution and 228 nm for the fifth dilution which indicated that the nanoparticles were photosensitive and the SEM study confirmed the spherical nature of these nanoparticles. The total synthesis time was approximately 5 h after drying the moringa leaves, and the average particle size was approximately 16 nm. Such synthesized nanoparticles can potentially be useful for drug delivery, especially in Low and Middle Income Countries (LMICs).

## Introduction

There are quite limited green synthesis studies of Fe_3_O_4_-NPs via biological routes and their use in the biomedical field, especially in LMICs [1]. Table 1 indicates the size and morphology of magnetite crystals which play an important role in influencing magnetite's properties [2]. Interestingly, Fe_3_O_4_-NPs are biocompatible, biodegradable, and potentially nontoxic to humans [3]. These properties contribute to the versatility of Fe_3_O_4_-NPs and show great potential in future biomedical applications such as targeted drug delivery, antibacterial, tissue engineering, and so on. In this regard, numerous Fe_3_O_4_-NP synthesis methods, for example, coprecipitation, the sol–gel method [4], hydrothermal synthesis [5], solid-state synthesis [6], flame spray synthesis [7], thermal decomposition [5], and solvothermal methods [8], have been adopted to produce nanoparticles with desired properties. However, such methods have had a number of limitations, including high production costs, toxic chemicals, and the production of hazardous byproducts [9–12]. This has necessitated research in green synthesis approaches in an effort to address the above issues caused by these conventional methods [13]. Green synthesis has many advantages, such as being simple, having fast manufacturing procedures, having lower production costs, and producing less waste [14].

**Table 1:** Different green synthesized plant parts with their corresponding morphologies

Plant name	Plant part	Synthesized size	Morphology	References
Fruit peels	Plantain peel	30–50 nm	Spherical	[15]
	Punica Granatum (pomegranates)	Diameter = 40 nm Length = above 200 nm	Rod	[16]
	Rambutan	100–200 nm	Agglomerated, spinel	[17]
	Ananas comosus	10–16 nm	Agglomerated, spherical	[18]
	Citrullus lanatus	Less than 17 nm	Agglomerated, spherical	[19]
	Citrus aurantium	17–25 nm	Slightly elongated	[20]
	Punica granatum	–	Slightly rod-shaped	[20]
	Malus domestica	–	Spherical	[20]
	Citrus limon	–	Spherical	[20]
Fruit	Passiflora tripartita (Banana passionfruit)	18.2–24.7 nm	Spherical	[21]
	Averrhoa carambola	1.9–3.1 nm	Spherical	[22]
	Lemon	14–17 nm	Spherical	[23]
	Couroupita guianensis	17 ± 10 nm	Spherical	[24]
Leaf	Carob	4–8 nm	Well monodisperse	[25]
	Tridax procumbens	–	Irregular shape—rough surfaces	[26]
	Artemisia annua	3–10 nm	Spherical	[27]
	Caricaya papaya	33 nm (from XRD)	Agglomerated plate like structure with coarsened grains and capsule like	[28]
	Perilla frutescens	Approx. 50 nm	Spherical	[27]
	Euphorbia wallichii	10–15 nm	Spherical	[29]
	Green tea	5.7 ± 4.1 nm	Spherical	[30]
	Zea mays L. (ear leaves)	–	Aggregated spherical	[31]
	Sesbania grandiflora	25–60 nm	Agglomerated nonspherical	[32]
	Rubus glaucus Benth	40–70 nm	Aggregated spherical	[33]
	Calliandra haematocephala	Approx. 85.4– 87.9 nm	Bead-like spherical	[34]
	Lagenaria siceraria	30–100 nm	cubical	[35]
Seed	Grape seed proanthocyanidin	Approx. 30 nm	Irregular shape	[36]
	Syzygium cumini	9–20 nm	Agglomerated spherical	[37]
Plant	Soya bean sprouts	Approx. 8 nm	Spherical	[38]
	Aloe vera	93–227 nm	Spherical	[39]
	Aloe vera	Approx. 6–30 nm	Agglomerated irregular	[40]
Marine plant	Sargassum muticum (Japanese weed)	18 ± 4 nm	Cubical	[41]
	Kappaphycus alvarezii	14.7 ± 1.8 nm	Spherical	[42]
	Padina pavonica	10–19.5 nm	Spherical	[43]
	Sargassum acinarium	21.6–27.4 nm	Spherical	[43]
Root	Mimosa pudica (sensitive grass)	60–80 nm	Agglomerated rough spherical	[44]
Stolon	Potato	40 ± 2.2 nm	Cubic	[45]
Waste	Tea residue	5–25 nm	Cuboid/pyramid	[46]
	Rice straw	9.9 ± 2.4 nm	Aggregated spherical	[47]
	Coffee waste hydrochar	10–40 nm	Spherical	[48]
	Acacia mearnsii (biochar)	18–35 nm	Uneven	[49]
Gum	Arabic gum	70–80 nm	Spherical	[50]

Medicinal plants can easily be conjugated with Fe_3_O_4_-based nanoparticles to produce drug delivery applications [51]. This is because of their ability to produce excellent formulations that yield to multiple biological signaling pathways. Among the many plants that have inspired green synthesis is Moringa oleifera (MO) [52]. MO was initially used in the treatment of inflammation, cancer, bacterial/viral infections and hyperglycaemia because of its high bioactive and antioxidant compounds. MO is excellently rich in such polyphenols and provides a wonderful synthesis agent for the necessary nanoparticles [53]. Regarding anticancer potential, Moringa oleifera (MO) has the ability to fight various cancers [54]. However, it seems challenging to produce such Fe_3_O_4_-based nanoparticles using MO.

The aim of this study is therefore to develop an appropriate protocol for green biosynthesis and characterization of Fe_3_O_4_-NPs using MO leaves given the multiple drug delivery applications from such particles. It was hypothesized that such green synthesized nanoparticles may greatly be applicable in targeted drug delivery especially during cancer treatment. Based on the researchers’ knowledge, this is the first attempt to use Ugandan grown MO for green synthesis and characterization of iron oxide nanoparticles.

## Main text

### Materials

Ferrous iron (III) chloride (FeCl_3_) was of analytical grade and purchased from Smakk International Ltd., a laboratory supplies company in Kampala. This chloride was additionally used without further purification and was dissolved into deionized (DI) water for all the synthesis procedures. MO leaves were collected from a Moringa plantation found in Eastern Uganda.

### Preparation of MO leaves into MO extract solution

**Fig. 1:**
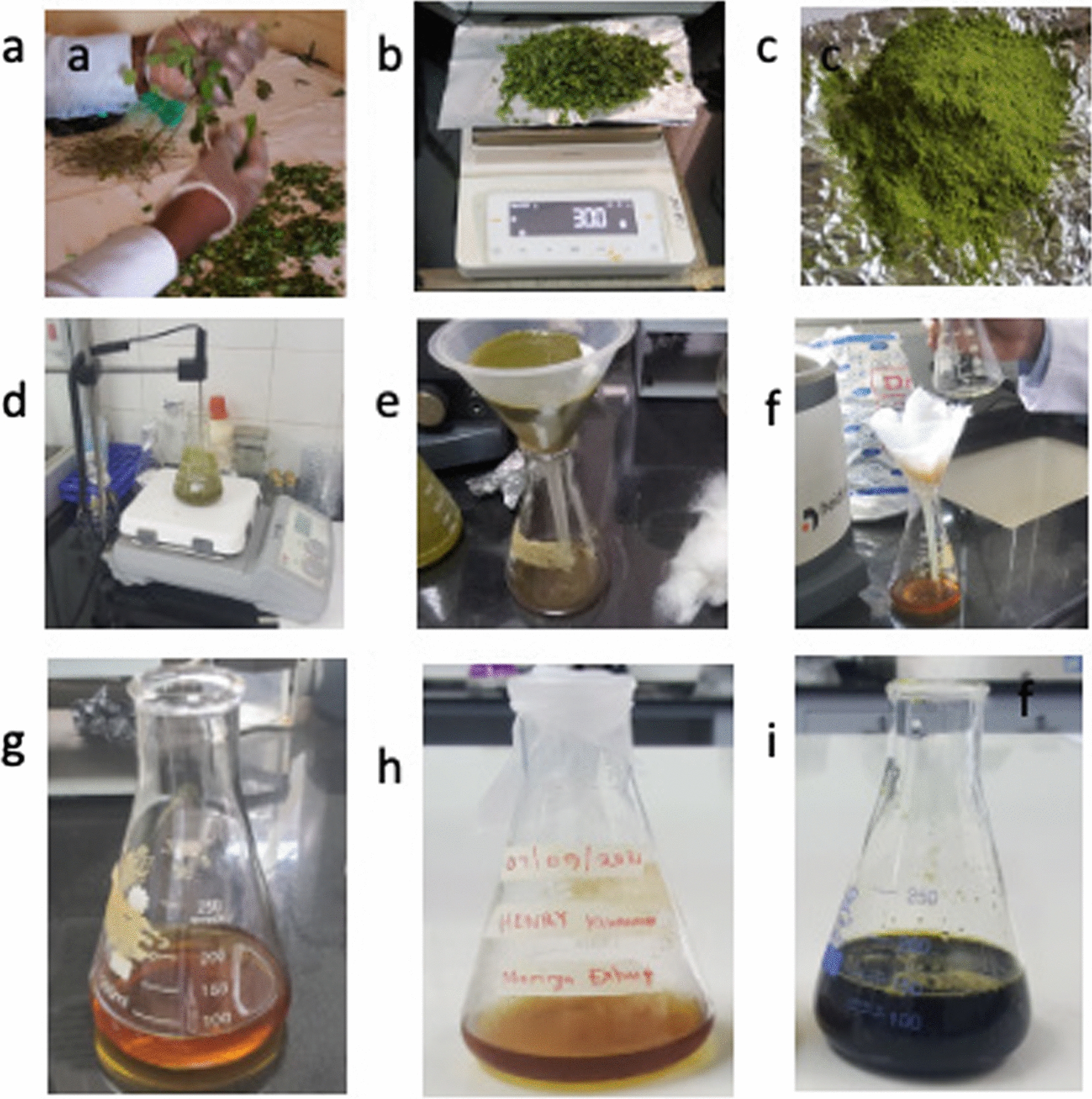
The extraction process of moringa solution from moringa leaves **a** Sorting and cleaning **b** Weighing the sample **c** Grinded powder sample **d** Heating the sample **e** Cotton wool filtered MO extract **f** Nylon filtered MO extract **g** Fe3Cl4 solution **h** MO extract **i** MO-Fe3Cl4 solution

MO leaves were hand sorted and dried under room temperature for 72 h as per Fig. 1. 30 g of the dried leaves were then measured using a sartorius measuring scale (Max 5200, Germany) and ground using a silver crest powder grinder (SC-1880) at a rotating speed of 28,000 revolutions per minute for 5 min. 10 g of Moringa powder was mixed with 100 ml of DI water in an Erlenmeyer flask and heated at 80 °C while stirring using a magnetic stirrer for 1 h at a rate of 200 revolutions/per minute. The heated moringa solution was allowed to cool for 3 h and then filtered initially using cotton wool and then nylon filter to obtain a fine moringa solution, as shown in Fig. 1f. All this work was done from the Research Center for Tropical Diseases and Vector Control (RTC) of Makerere University College of Veterinary, Animal Resources and Biosecurity (COVAB).

### Preparation of the Moringa oleifera-iron(III) chloride (MO-Fe_3_Cl_4_) solution

Following a protocol from Aisida et al. [55], 0.6 M of Iron(III) chloride solution was prepared by mixing ferrous Iron(III)chloride with 100 ml of DI water and shaken to fully dissolve for approximately 15 min. 80 ml of this iron(III)chloride solution was mixed with 20 ml of the MO solution to form the MO-Fe_3_Cl_4_ solution. Deviating a bit from this protocol, this solution was placed in a water bath at 60 °C and was allowed to run for 4 h to activate the phytochemicals. This solution was cooled for 2 h at room temperature and thereafter stored in a refrigerator at 4 °C for future use.

### Preparation of Moringa oleifera-Iron(III)chloride (MO-Fe_3_Cl_4_) dilutions for UV–Vis analysis

Different MO-Fe_3_Cl_4_ solutions were prepared using a serial dilution procedure to clearly space and characterize the suspected particles using a UV–visible spectrometer [56, 57]. Six dilutions were obtained with the first one obtained by mixing 2 ml of DI water into 1 ml of MO-Fe_3_Cl_4_ solution. The second dilution was obtained by mixing 1 ml of the first dilution with 2 ml of DI water, the third was obtained by mixing 1 ml of the second dilution with 2 ml of DI water, the fourth was obtained by mixing 1 ml of the third dilution with 2 ml of DI water, the fifth was obtained by mixing 1 ml of the fourth dilution with 2 ml of DI water and finally the sixth was obtained by mixing 1 ml of the fifth dilution with 2 ml of DI water. The DI water graph was used as a control graph to clearly isolate the peaks obtained from this solvent in comparison with those obtained from the MO-Fe_3_Cl_4_ solution. Farther dilutions never showed any difference in the UV–Vis graph, hence ending with the fifth dilution.

### Characterization of the nanoparticles

The synthesized nanoparticles were characterized by using a UV–Vis, XRD, SEM, and EDX. The optical properties of the synthesized nanoparticles were examined and confirmed using a double beam UV–Vis (Jenway 6715, UK) using a spectral range of 200–400 nm from Makerere University’s RTC lab. A powder XRD employing a Bruker AXS diffractometer, (Bruker, Germany) and fitted with Cu-Ka radiation (λKα_1_ = 1.5406 Å) from 2θ = 0.5°–130°, with increments of Δ2ϑ: (0.034°), voltage of 40 kV, current of 40 mA, power of 1.6KW, and counting time of 0.5 s/step was used to analyze approximately 500 mg of green synthesized Fe_3_O_4_-NPs powder. This was done from the Materials Research Department (MRD), iThemba LABs, Cape Town in South Africa. The generated data were analysed by OriginPro, and the resultant peaks and two theta values were compared with the standard Fe_3_O_4_-NP values from the International Center for Diffraction Data (ICDD) database. The structural morphology of the prepared nanoparticles was determined by a ZEISS (Gemini 1, Germany) scanning electron microscope and EDX from Makerere University’s Mechanical Engineering Department at a working distance (WD) of 7.9 mm and an accelerating voltage of 10 kV under vacuum conditions.

### Results and discussion

The results below indicate the characteristics of the produced nanoparticles.

#### UV–Vis analysis

The formation of nanoparticles was evidenced by the appearance of an instantaneous dark black color change from brown in the solution, as shown in Fig. 1i. This formation was due to a variety of plant biomolecules (polyphenols), which played a major role in the reduction of metal ions and sufficiently stabilized the Fe_3_O_4_-NPs. Phytochemicals bound to the surface of these nanoparticles are rich in hydrophilic hydroxyl groups that allow the NPs to disperse and distribute homogenously in aqueous solutions [58]. Thus, after the reaction, it can be seen that the UV spectra of the fabricated nanoparticles had absorption bands at lower concentrations than at higher concentrations.

The UV–Vis absorption peaks (225 nm and 297 nm) are also attributed to the presence of alkaloids, phenolic acids, flavonoids, tannins, terpenoids and carbohydrates in the MO aqueous extract. The DI water and the sixth dilution clearly indicate both peaks compared to other graphs [59]. This was evidenced by a 268 nm absorption peak that was produced by the DI water graph, confirming the occurrence of a synthesis process.

**Fig. 2:**
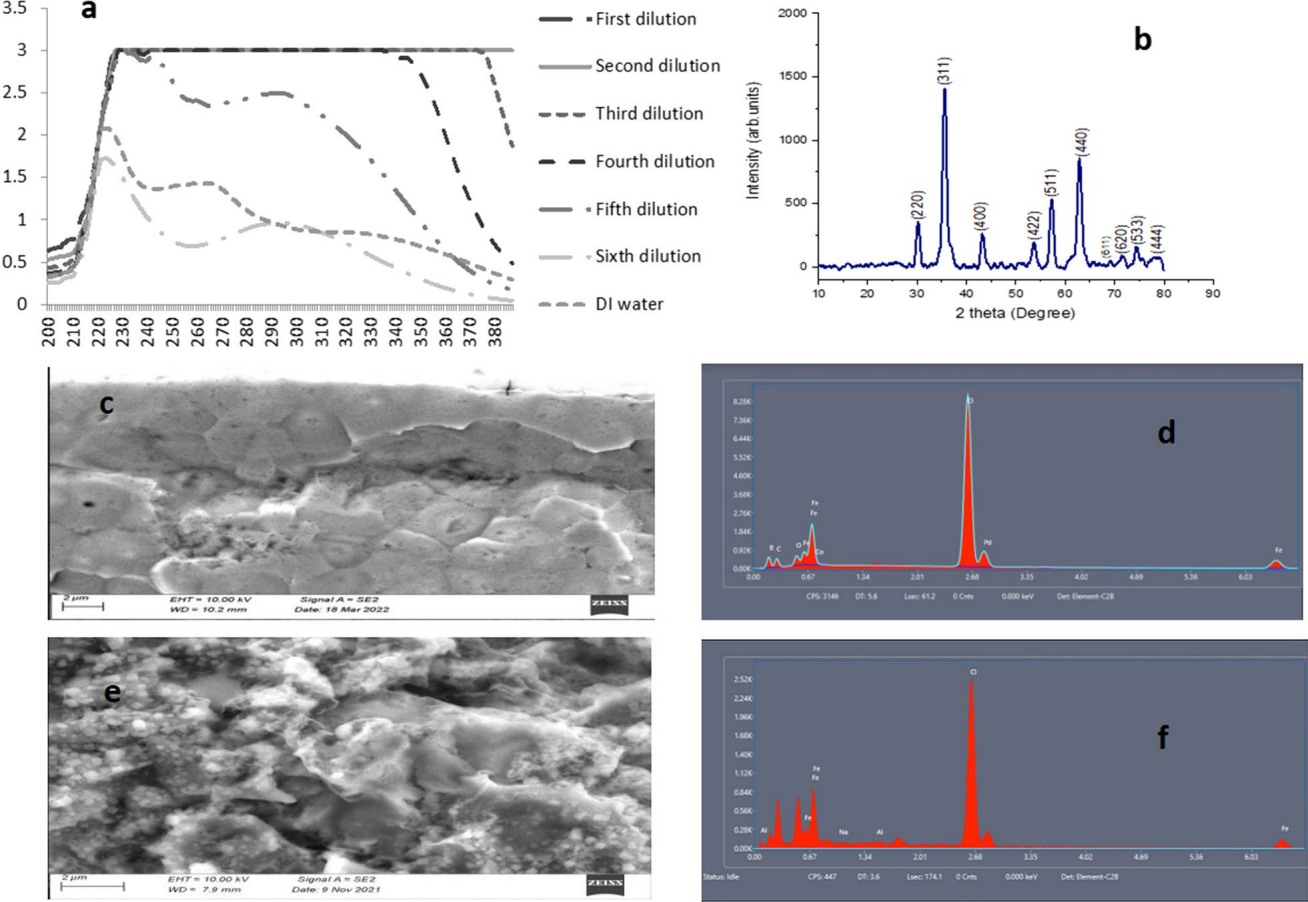
**a** UV–Vis graphs showing different dilutions **b** XRD graph for the Iron-oxide biosynthesized Fe_3_O_4_-NPs **c** SEM image for the iron (III) chloride precursor **d** XRD for the iron (III) chloride precursor **e** SEM image for the Iron-oxide biosynthesized Fe_3_O_4_-NPs **f** XRD for the Iron-oxide biosynthesized Fe_3_O_4_-NPs

Additionally, the UV–Vis results showed a maximum absorption peak at 225 nm for the sixth dilution and 228 nm for the fifth dilution, followed by the peak at 297 nm for both dilutions. This could be due to the excitation of nanoparticles from the ground to the excited state [60]. The high concentration of leaf extract enhanced the phytochemical content of the extract, which reduced the precursor quickly, leading to rapid nanoparticle formation that enhanced the absorbance value, as shown in Fig. 2a [61]. Therefore, the UV–Vis analysis concluded that Fe_3_O_4_-NPs had an intense absorbance at ∼300 nm, hence indicating the photosensitivity of the synthesized particles in the UV region [62].

#### XRD analysis

XRD analysis generated ten peaks for the biosynthesized Fe_3_O_4_-NPs positioned at 2θ angles of 30.2°, 35.5°, 43.2°, 53.8°, 57.3°, 62.95°, 69.0°, 71.4°, 74.3°, and 78.1°. The observed lattice spacings at 30.2°, 35.5°, 43.2°, 53.8°, and 57.3° matched well with the (220), (311), (400), (422), and (511) planes of Fe_3_O_4_ crystals (Fig. 2b). The crystal structure data was in close agreement with the reported data and can be assigned to the magnetite phase of iron oxide [63]. This XRD pattern for magnetic nanoparticles is cross referenced with ICDD—International Centre for Diffraction Data (ICDD) file number: 00–019-0629. The peak intensity ranged from 240 to 1,400 arbitrary units for the synthesized Fe_3_O_4_-NPs.

#### Scanning electron microscope and energy dispersive X-ray analysis

Figure 2c never indicated the synthesized Fe_3_O_4_-NPs as compared to Fig. 2e. This clearly confirmed that such nanoparticles were a reaction result between MO and Iron(III) chloride precursor. Fe_3_O_4_-NPs exhibited a granular, homogenous, spherical-shaped structure with an average diameter of approximately 16 nm. Given the unique atomic structure of each element, EDX was additionally used to provide information about the chemical composition of each element as it interacts between the X-rays and the compound being investigated. Therefore, when this analysis was carried out, the X-rays reflected off the iron compound to give peak amplitudes that helped to identify the elements present in the compound being studied. The peak amplitude of iron started from approximately 0.66 to 7 keV, as shown by Fig. 2d and f which confirmed the presence of the iron elements in the compounds using EDX [64]. The results also demonstrated the high percentage of iron present in the particles, as the EDX spectra revealed the presence of iron peaks in three different areas (0.66, 0.68 and 7.0). Energy dispersive X-ray spectroscopy (EDX) was also used to confirm iron oxide nanoparticle formation and obtain more structural details about the suspension. There were several peaks of Fe with other elements, such as sodium, aluminium and chlorine, thus indicating the ability for organic materials to be used as capping agents.

#### Energy dispersive X-ray analysis

EDX analysis further provided the qualitative and quantitative status of the elements, which may have affected the fabrication of the NPs. This analysis showed that the EDX spectrum contained intense peaks of Cl and Fe in addition to minor peaks of Na and Al. The Fe and Cl peaks might have originated from the FeCl_3_ precursors used in the fabrication of these nanoparticles. The Na and Al peaks could mainly have been due to the polyphenol groups or other sodium/aluminum-containing biomolecules present in the MO leaf extract. The higher percentages of Cl indicated the plant biomolecules presence in the metal ions reduction and stabilization of the nanoparticles. These values might also be helpful in observing the atomic content on the surface and near the surface region of the produced nanoparticles. Such nanoparticles can potentially be used in cancer [65], bacterial [66] and viral [67] treatment mechanisms that greatly affect LMICs.

## Conclusion

A novel green synthesis of iron oxide nanoparticles using Ugandan grown MO has been demonstrated. This first time trial of nanoparticle formulation has been confirmed by SEM to have a spherical shape with a 16 nm particle size. Given no requirements for extra surfactants or reductants, this method can serve as a simple and eco-friendly protocol for use in LMICs.

## Limitations

The following studies would have confirmed our results better but could not be done due to limited resources: 1. Fourier transform infrared (FTIR) analysis of the nanoparticles, 2. Vibrating sample magnetometry studies, 3. Cancerous cell viability studies.

## Data Availability

The raw data analysed during the current study is available from the corresponding author on reasonable request.

## Abbreviations

MO: Moringa oleifera
LMICs: Low- and middle-income countries
Fe_3_O_4_: Iron (III) Oxide
Fe_3_O_4_: NPs Iron (III) Oxide nanoparticles
UV–Vis: Ultraviolet visible
SEM: Scanning electron microscope
XRD: X-ray diffraction
EDX: Energy dispersive X-ray
Fe_3_O_4_: Iron oxide
NPs: Nanoparticles
DI: Deionized
WD: Working distance
MRI: Magnetic resonance imaging
RTC: Research center for tropical diseases and vector control
ICDD: International center for diffraction data
FTIR: Fourier transform infrared
nm: Nanometers
